# FIT AND STRONG! IN ILLINOIS: LESSONS LEARNED

**DOI:** 10.1093/geroni/igad104.2844

**Published:** 2023-12-21

**Authors:** Susan Hughes, Andrew DeMott, Julie Bobitt

**Affiliations:** University of Illinois at Chicago, Chicago, Illinois, United States; University of Illinois at Chicago, Chicago, Illinois, United States; University of Illinois at Chicago, Chicago, Illinois, United States

## Abstract

This session will describe lessons learned from a major effort to disseminate Fit & Strong! (F&S) statewide in Illinois, a state with both strong urban and rural counties. F&S is an evidence-based program for older adults who experience lower extremity mobility challenges. It is recommended for dissemination by the Administration on Community Living and the Centers for Disease Control and Prevention and is currently offered in 32 states. We received a substantial ARPA contract from the Illinois Department of Public Health (IDPH) to disseminate F&S sustainably throughout Illinois, between July 2022 and June 2023. We have convened Referral Network and Sustainability Workgroups to guide the work. Our goal is to identify 111 community-based organizations (CBOs) who can enroll 1,667 older adults into the program. We have developed an extensive number of collaborators including the aging network and a large number of healthcare systems and plans. We are also collaborating with the Illinois Extension Program to reach underserved rural areas of the state. This extensive collaborative effort has enabled us to enroll 70 CBOs that plan to offer the program. This collaboration between aging and healthcare networks that is co-sponsored by the Illinois Department on Aging and IDPH appears to be enabling us to reach a considerable number of providers who previously were not aware of or offering F&S. We are collecting pre/ post participant outcomes to evaluate program impact and examining state fall rates and uptake of lower extremity joint replacements. Lessons learned and preliminary findings will be discussed.

